# Supplementary figures and images for: The small RNA landscape is stable with age and resistant to loss of dFOXO signaling in Drosophila
Source: PLoS One. 2022 Nov 16;17(11):e0273590. doi: 10.1371/journal.pone.0273590 (PMC9668163; doi:10.1371/journal.pone.0273590)

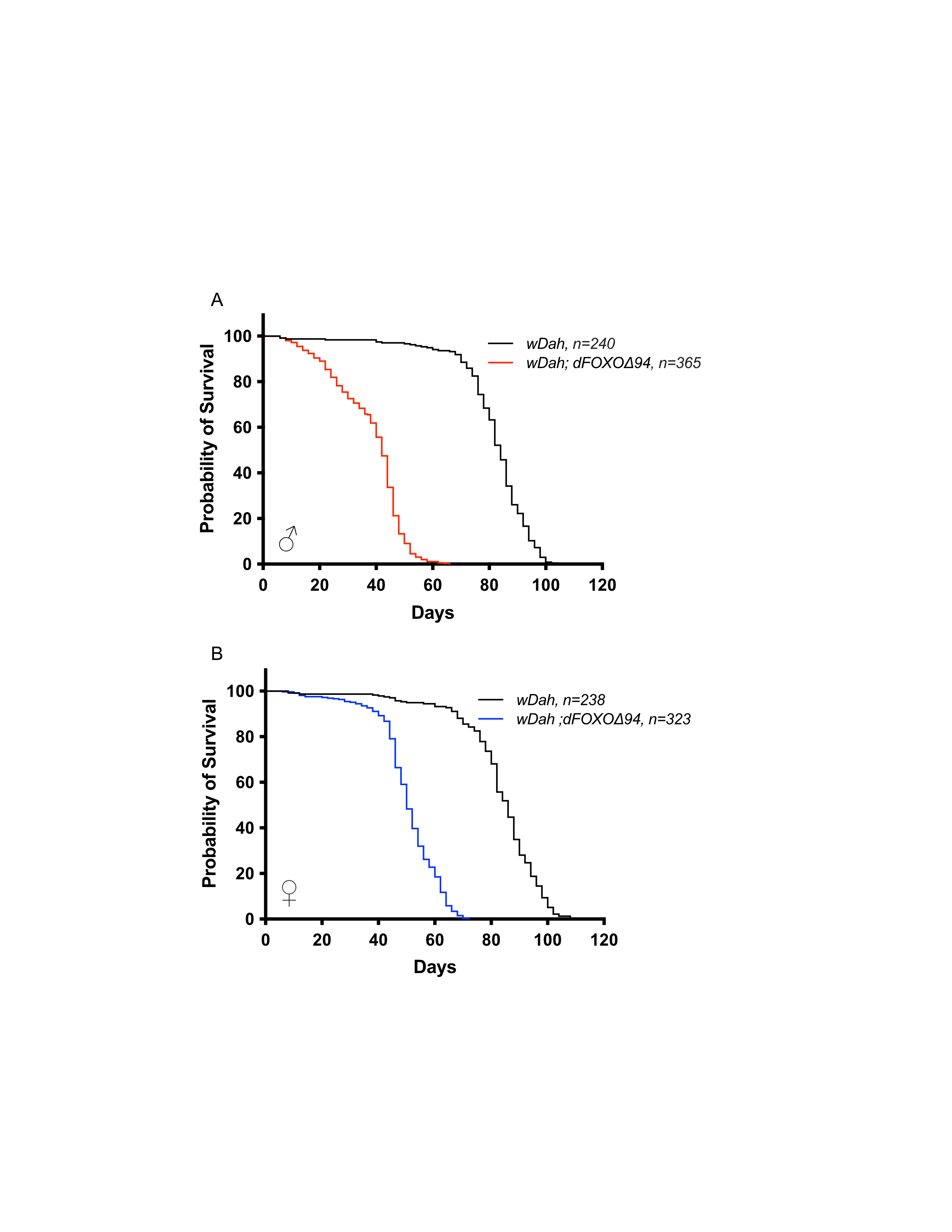

Supplement: S1 Fig — Percent survival of wildtype (wDAH) and dFOXO-null (wDAH; foxoΔ94) flies were recorded 2–3 times weekly. (A) Mated wildtype males had a median lifespan of 90 days and a maximum survival of 105 days while mated dFOXO-null males had a median lifespan of 45 days and a maximum survival of 65 days. (B) Mated wildtype females had a median lifespan of 90 days and a maximum survival of 110 days while mated dFOXO-null females had a median lifespan of 55 days and a maximum survival of 70 days. (TIFF) [file pone.0273590.s001.tiff]

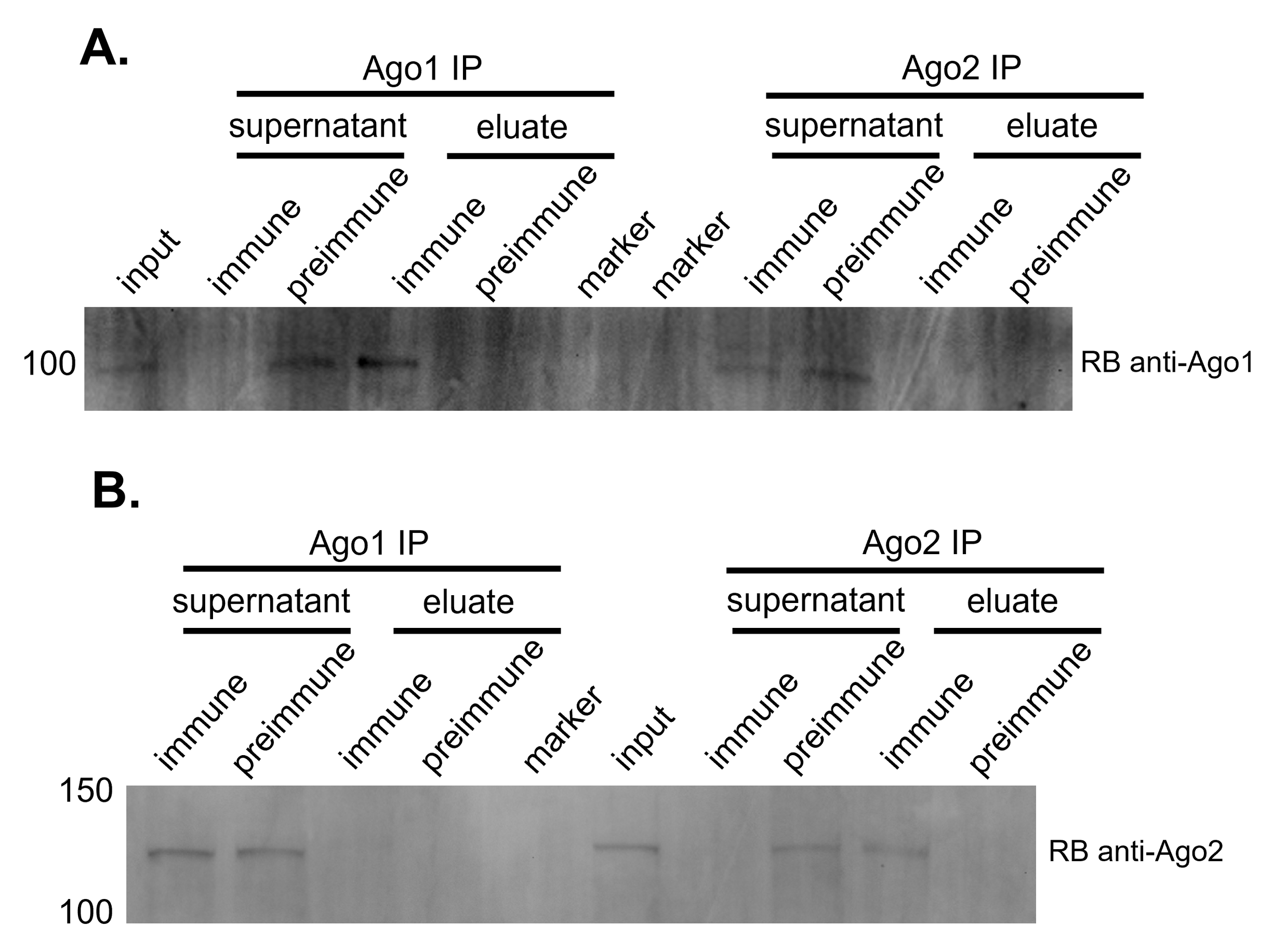

Supplement: S2 Fig — (A) Guinea pig anti-Ago1 antisera, Guinea pig anti-Ago2 antisera, or preimmune antisera were used in an immunoprecipitation (IP) using lysate from Drosophila melanogaster Schneider 2 cells as input. Western blot of input, supernatant, and eluate was probed with a commercial rabbit (RB) anti-Ago1 antibody. Anti-Ago1 antisera immunoprecipitated Ago1 and was capable of depleting the lysate of Ago1. Preimmune sera and anti-Ago2 antisera did not immunoprecipitate Ago1. (B) A second blot was probed with a commercial rabbit anti-Ago2 antibody. Similarly, anti-Ago2 antisera was able to immunoprecipitate Ago2 and depleted the lysate of Ago2. Preimmune sera and anti-Ago1 antisera did not immunoprecipitate Ago2. (TIF) [file pone.0273590.s002.tif]

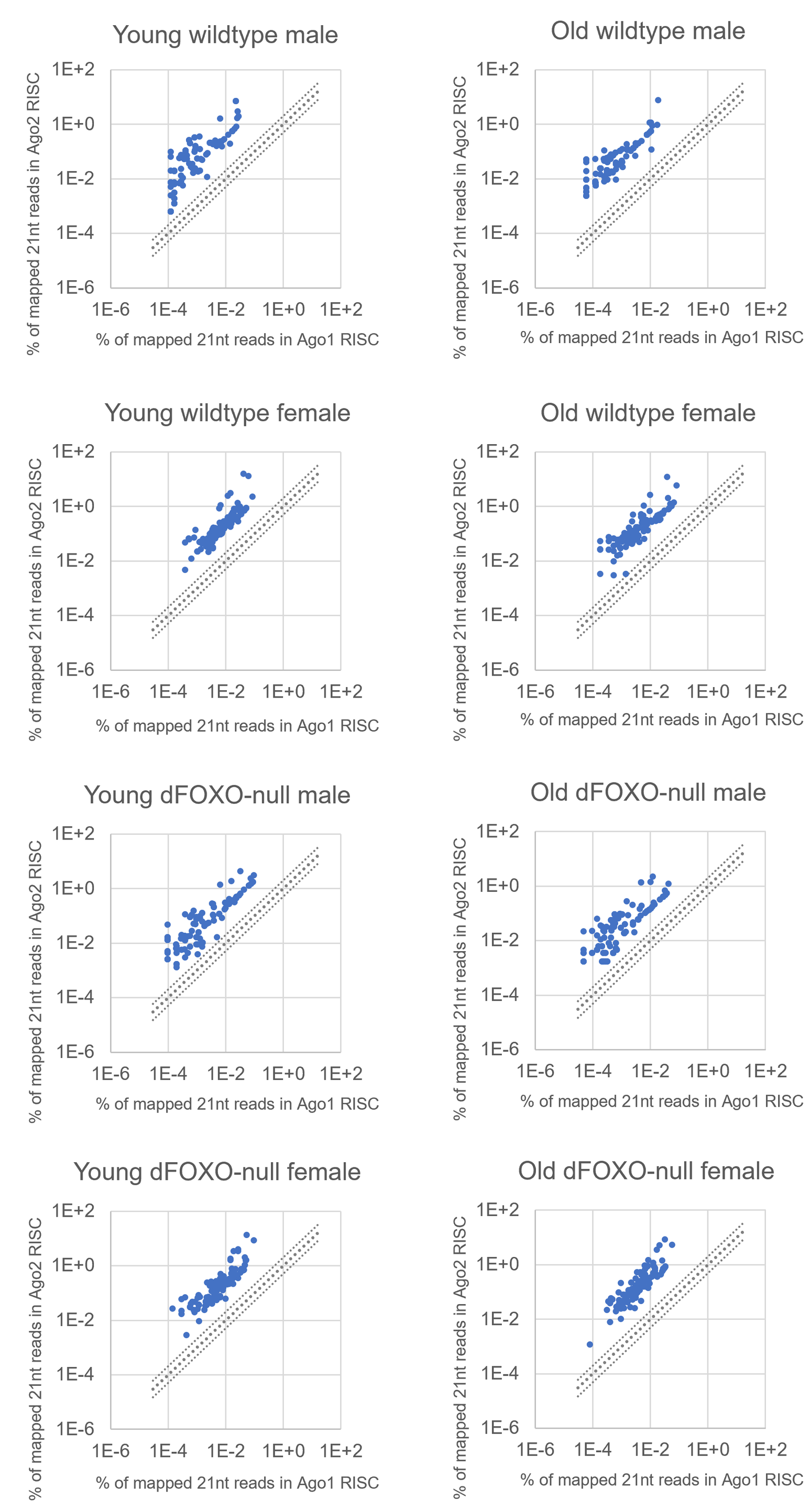

Supplement: S3 Fig — Small RNA immunoprecipitated with Ago1 RISC or Ago2 RISC from young (5 day old) or old (35 day old) whole flies were sequenced, size selected to 21 nt in silico, and mapped to known Drosophila miRNAs and transposons (n = 2). Transposon siRNAs are plotted as percent of mapped reads in Ago1 RISC against percent of mapped reads in Ago2 RISC. All transposon siRNAs that were detected in both RISC make up a greater percentage of Ago2 RISC. (TIF) [file pone.0273590.s003.tif]
