# Supplementary material for: The small RNA landscape is stable with age and resistant to loss of dFOXO signaling in Drosophila
Source: PLoS One. 2022 Nov 16;17(11):e0273590. doi: 10.1371/journal.pone.0273590 (PMC9668163; doi:10.1371/journal.pone.0273590)
Supplement: S1 Table — Small RNAs from whole flies, Ago1 RISC immunoprecipitation (IP), or Ago2 RISC IP were sequenced, mapped to known Drosophila miRNAs and transposons, and normalized as a percent of total reads mapped (n = 2). miRNA reads were then normalized by total miRNA reads and shown here. (A) The 20 most abundant miRNAs in young wildtype males make up 87 to 94 percent of the total miRNA reads in the male libraries. (B) The 20 most abundant miRNAs in young wildtype females make up 79 to 87 percent of the total miRNA reads in the female libraries. (PDF) [file pone.0273590.s004.pdf]

S1A Table. 20 miRNAs make up the majority of miRNAs detected in total small RNA, Ago1, and Ago2 RISC.												
	Male											
	Wildtype						dFOXO-null					
	Total abundance		Ago1 RISC		Ago2 RISC		Total abundance		Ago1 RISC		Ago2 RISC	
	Young	Old	Young	Old	Young	Old	Young	Old	Young	Old	Young	Old
miR-276a-3p	25.2	20.8	17.2	13.9	19.8	18.0	23.3	17.4	16.7	16.9	19.9	14.8
miR-276b-3p	16.4	14.1	13.3	10.5	15.3	13.3	15.4	12.1	12.5	12.1	14.3	11.4
miR-1-3p	15.4	9.5	22.5	11.9	16.0	11.2	18.7	10.1	18.3	12.9	15.6	10.8
bantam-3p	8.0	7.8	5.1	4.8	6.0	5.4	7.1	7.6	5.7	4.3	5.0	4.1
miR-8-3p	4.1	4.6	3.5	6.0	3.6	4.5	4.1	5.9	4.5	5.0	3.0	5.3
miR-956-3p	2.7	3.7	3.0	4.7	3.2	4.9	3.0	3.6	2.6	3.0	3.0	2.9
miR-999-3p	2.6	1.6	2.0	1.3	1.5	1.2	2.4	1.4	1.6	1.3	1.2	0.8
miR-277-3p	2.4	2.9	4.5	4.5	4.6	3.6	2.4	3.0	4.2	2.3	4.1	3.6
miR-34-5p	2.2	6.8	3.0	7.9	4.2	6.1	1.9	6.7	4.5	8.6	5.1	11.2
miR-184-3p	2.1	2.2	3.1	3.0	2.7	2.8	2.7	2.3	2.9	3.9	3.3	3.0
miR-263a-5p	1.9	2.0	1.5	1.8	1.5	2.5	1.9	1.9	1.1	1.5	1.3	1.5
miR-9a-5p	1.9	1.9	0.6	0.5	0.5	0.8	1.9	2.1	0.4	0.6	0.4	0.5
miR-10-3p	1.8	1.9	1.8	1.8	2.0	2.2	2.2	2.9	1.8	2.5	2.2	2.6
miR-7-5p	1.7	1.4	0.8	0.7	1.0	1.0	1.6	1.3	0.6	0.6	0.7	0.7
miR-14-3p	1.1	5.2	7.7	13.2	4.8	5.8	0.5	6.7	10.9	10.4	7.3	10.1
miR-375-3p	1.0	0.9	0.6	0.5	0.6	0.5	1.1	1.1	0.5	0.6	0.6	0.5
miR-278-3p	0.9	1.5	1.4	1.4	1.5	1.4	0.8	1.6	1.3	2.1	1.3	1.4
miR-31a-5p	0.8	0.7	0.4	0.4	0.4	0.5	1.0	0.7	0.3	0.4	0.4	0.4
let-7-5p	0.7	0.8	0.2	0.2	0.2	0.2	0.7	0.9	0.2	0.2	0.2	0.2
miR-11-3p	0.7	1.0	0.6	0.9	0.7	1.0	0.6	1.1	0.5	0.8	0.5	0.9
**Total of top 20 miRNAs**	**93.6**	**91.1**	**92.6**	**90.0**	**89.7**	**86.9**	**93.1**	**90.3**	**91.0**	**90.0**	**89.3**	**86.5**
Other miRNAs	6.4	8.9	7.4	10.0	10.3	13.1	6.9	9.7	9.0	10.0	10.7	13.5

**S1B Table. 20 miRNAs make up the majority of miRNAs detected in total small RNA, Ago1, and Ago2 RISC.**												
	Female											
	Wildtype						dFOXO-null					
	Total abundance		Ago1 RISC		Ago2 RISC		Total abundance		Ago1 RISC		Ago2 RISC	
	Young	Old	Young	Old	Young	Old	Young	Old	Young	Old	Young	Old
miR-276a-3p	18.7	17.1	16.1	16.4	15.7	16.2	19.0	22.1	15.8	18.2	16.7	16.2
miR-1-3p	14.8	7.9	19.9	14.4	12.1	9.6	15.3	3.1	15.2	8.5	11.5	8.8
miR-276b-3p	12.1	10.4	12.0	12.1	11.5	11.8	12.4	14.8	11.2	12.8	11.4	11.7
bantam-3p	10.7	14.5	7.3	5.4	6.4	5.0	10.1	10.2	6.3	6.3	7.6	5.1
miR-8-3p	5.1	3.3	3.1	3.5	5.1	6.2	4.4	2.7	4.3	4.3	5.2	6.5
miR-956-3p	2.8	5.6	4.0	5.8	6.5	5.8	4.9	11.9	5.5	5.9	6.0	7.1
miR-318-3p	2.4	0.8	2.8	0.8	1.6	0.4	1.6	0.8	1.5	1.0	0.9	0.5
miR-10-3p	2.3	2.6	1.6	2.1	2.6	3.3	2.0	1.3	1.6	2.3	2.2	3.3
miR-999-3p	2.3	1.3	1.2	1.1	1.0	1.0	1.7	0.7	1.0	0.6	1.2	0.8
miR-184-3p	2.3	2.6	2.9	3.2	2.3	2.5	3.3	1.2	4.8	6.3	3.9	3.6
miR-277-3p	2.2	1.4	3.6	3.1	2.8	2.8	1.5	2.9	3.0	1.7	2.5	1.8
miR-9b-5p	1.9	1.0	1.1	1.0	0.6	0.6	1.9	1.4	1.2	0.8	1.0	0.7
miR-7-5p	1.8	1.6	0.7	0.8	0.6	0.7	1.4	0.7	0.6	0.6	0.6	0.6
miR-9a-5p	1.7	1.4	0.4	0.5	0.3	0.4	1.5	1.0	0.3	0.2	0.4	0.3
miR-34-5p	1.6	6.1	3.4	5.7	3.2	5.3	0.9	2.8	3.5	6.0	3.4	5.1
miR-994-5p	1.6	0.5	1.0	0.2	3.8	0.6	1.7	0.8	0.8	0.4	2.2	1.2
miR-263a-5p	1.2	1.7	0.9	1.4	1.9	2.5	1.1	0.6	0.9	1.3	1.2	1.8
miR-14-3p	1.1	2.9	4.7	7.3	2.7	3.2	0.3	2.4	6.0	5.7	2.4	2.2
miR-278-3p	0.9	1.7	1.3	1.4	1.1	1.0	1.4	4.0	1.5	2.8	1.4	1.6
miR-9c-5p	0.9	0.6	0.3	0.3	0.5	0.5	1.0	1.2	0.3	0.3	0.5	0.4
**Total of top 20 miRNAs**	**88.1**	**85.0**	**88.0**	**86.3**	**82.3**	**79.3**	**87.2**	**86.4**	**85.3**	**86.0**	**82.0**	**79.2**
Other miRNAs	11.9	15.0	12.0	13.7	17.7	20.8	12.8	13.6	14.7	14.0	18.0	20.8
