# Supplementary material for: The small RNA landscape is stable with age and resistant to loss of dFOXO signaling in Drosophila
Source: PLoS One. 2022 Nov 16;17(11):e0273590. doi: 10.1371/journal.pone.0273590 (PMC9668163; doi:10.1371/journal.pone.0273590)
Supplement: S2 Table — Small RNAs from whole flies, Ago1 RISC immunoprecipitation (IP), or Ago2 RISC IP were sequenced, size selected in silico, mapped to known Drosophila miRNAs and transposons, and normalized as a percent of total reads mapped (n = 2). Transposon siRNA reads were then normalized by total transposon siRNA reads and shown here. (A) The 20 most abundant transposon siRNAs in young wildtype males make up 57 to 88 percent of the total transposon siRNA reads in male total small RNA and Ago2 RISC. (B) The 20 most abundant transposon siRNAs in young wildtype females make up 55 to 66 percent of the total transposon siRNA reads in the female total small RNA and Ago2 RISC. (PDF) [file pone.0273590.s005.pdf]

S2A Table. 20 transposon siRNAs make up the majority of transposon siRNAs detected in total small RNA and Ago2 RISC.												
	Male											
	Wildtype						dFOXO-null					
	Total abundance		Ago1 RISC		Ago2 RISC		Total abundance		Ago1 RISC		Ago2 RISC	
	Young	Old	Young	Old	Young	Old	Young	Old	Young	Old	Young	Old
297.0	44.4	50.9	7.5	11.4	29.0	44.0	3.9	7.1	0.7	1.3	5.0	10.6
roo	12.1	8.4	8.3	10.4	7.0	5.5	39.1	28.6	3.7	3.1	15.6	17.5
mdg1	6.3	4.4	9.3	7.1	8.0	5.0	10.3	11.4	11.0	11.0	11.0	9.4
opus	4.6	4.3	8.5	6.2	12.4	6.5	2.9	1.8	8.6	8.2	8.6	5.4
FB	4.4	5.1	2.1	5.6	6.6	6.6	5.9	8.1	1.8	2.6	6.6	11.1
accord2	2.0	0.9	4.7	6.5	0.8	0.7	2.3	5.2	2.4	3.4	1.1	2.0
blood	1.7	0.5	0.4	0.1	1.5	0.3	1.2	1.0	0.1	0.0	0.4	0.5
copia	1.5	2.6	1.2	1.9	0.8	1.1	1.0	1.6	0.4	0.6	0.4	1.6
gypsy	1.5	0.8	0.2	0.2	1.1	0.6	0.9	1.2	0.2	0.2	0.5	0.7
Stalker	1.1	0.6	0.3	0.3	0.6	0.4	2.3	0.9	0.4	0.3	1.0	0.7
rover	1.1	0.7	0.2	0.3	0.8	0.4	1.1	1.1	0.1	0.0	0.6	0.2
Stalker4	1.1	0.6	0.3	0.3	0.7	0.4	2.0	0.9	0.4	0.3	1.0	0.7
1360.0	1.0	0.8	2.5	1.8	0.6	0.4	1.4	1.2	0.9	1.2	0.3	0.6
Doc	0.8	1.3	1.0	0.9	0.9	1.0	2.0	2.2	1.5	1.3	1.5	1.5
Stalker2	0.8	0.3	0.1	0.1	0.4	0.3	1.5	1.1	0.4	0.1	0.8	0.4
gypsy4	0.8	0.7	0.1	0.2	0.5	0.4	0.9	0.9	0.0	0.2	0.4	0.8
F-element	0.8	0.8	0.9	0.9	0.9	0.7	1.2	1.5	1.1	1.1	1.1	1.1
17.6	0.7	0.9	1.4	0.9	1.0	0.7	0.4	0.4	1.5	1.7	1.0	0.8
Max-element	0.6	0.7	0.2	0.4	0.3	0.5	0.6	1.2	0.2	0.2	0.2	0.3
ninja	0.6	0.4	0.2	0.1	0.1	0.0	1.2	0.8	0.2	0.2	0.0	0.0
**Total of top 20 transposon siRNAs**	**88.0**	**85.7**	**49.3**	**55.6**	**73.9**	**75.7**	**82.0**	**78.0**	**35.6**	**36.8**	**57.0**	**65.9**
Other transposon siRNAs	12.0	14.3	50.7	44.5	26.1	24.3	18.0	22.0	64.4	63.2	43.0	34.1

S2B Table. 20 transposon siRNAs make up the majority of transposon siRNAs detected in total small RNA and Ago2 RISC.												
	Female											
	Wildtype						dFOXO-null					
	Total abundance		Ago1 RISC		Ago2 RISC		Total abundance		Ago1 RISC		Ago2 RISC	
	Young	Old	Young	Old	Young	Old	Young	Old	Young	Old	Young	Old
297.0	10.1	12.8	2.9	3.8	23.7	25.1	2.8	3.8	2.2	2.6	6.4	9.9
mdg1	8.3	8.7	4.3	8.0	19.7	12.5	10.5	12.6	4.4	3.9	21.6	15.8
roo	5.7	4.9	6.0	4.0	3.5	4.3	9.3	8.7	7.8	6.9	13.5	10.2
Max-element	4.7	4.8	2.6	1.8	1.0	0.9	3.8	4.0	3.3	3.4	1.1	1.2
F-element	4.0	3.4	3.3	1.9	1.2	1.1	3.8	3.1	3.4	3.4	1.2	1.4
Rt1b	3.8	4.1	2.5	1.0	0.9	0.7	4.6	5.3	2.6	2.8	0.9	0.8
GATE	3.3	3.1	2.2	1.1	0.8	0.6	3.1	2.6	2.2	2.1	0.6	0.7
R1A1-element	3.1	4.0	2.4	1.5	0.8	0.7	4.6	6.4	3.5	3.8	1.1	1.4
Stalker	2.9	2.3	3.6	2.2	1.4	0.7	3.4	2.6	4.2	4.1	2.6	1.6
Stalker4	2.7	2.1	3.6	2.0	1.3	0.7	3.2	2.4	4.0	4.0	2.5	1.6
rover	2.5	2.2	1.0	0.6	4.7	2.3	3.0	2.7	1.5	1.0	5.5	2.8
gypsy12	2.5	2.8	1.9	0.9	1.4	1.5	1.3	1.4	1.4	1.8	1.0	1.2
Doc	2.4	2.1	1.9	1.5	0.6	0.8	2.2	2.4	1.8	1.8	1.2	1.4
17.6	2.0	1.6	3.1	2.4	1.1	1.2	1.7	1.2	1.5	1.8	0.6	0.8
TART-C	2.0	0.9	0.8	0.7	0.4	0.4	0.9	0.8	1.0	1.0	0.4	0.3
Circe	1.5	1.0	1.3	0.7	0.7	0.7	0.9	0.8	1.0	1.1	0.5	0.5
invader6	1.4	1.2	1.2	0.5	0.9	1.1	0.7	0.5	0.8	0.9	0.4	0.5
opus	1.3	1.6	1.8	4.8	2.0	2.7	1.1	1.4	1.6	1.6	0.9	1.7
X-element	1.3	1.5	1.1	0.6	0.4	0.3	1.0	1.1	0.7	1.2	0.3	0.4
Stalker2	1.2	1.0	1.6	0.8	0.5	0.3	2.0	1.7	3.8	3.1	1.7	1.1
**Total of top 20 transposon siRNAs**	**66.4**	**66.0**	**49.0**	**40.6**	**67.1**	**58.4**	**63.8**	**65.5**	**52.5**	**52.5**	**64.0**	**55.3**
Other transposon siRNAs	33.6	34.0	51.0	59.4	32.9	41.6	36.2	34.6	47.5	47.6	36.0	44.7
