# Supplementary material for: The small RNA landscape is stable with age and resistant to loss of dFOXO signaling in Drosophila
Source: PLoS One. 2022 Nov 16;17(11):e0273590. doi: 10.1371/journal.pone.0273590 (PMC9668163; doi:10.1371/journal.pone.0273590)
Supplement: S1 Raw images — (PDF) [file pone.0273590.s006.pdf]

**RI1 Fig. Full blot of Ago1 Immunoprecipitation used in Fig2B.** Immunoprecipitation (IP) using guinea pig antisera against Ago1 (BRD-GP-3) or preimmune antisera was conducted on lysate from whole male flies and probed using (A) a commercial rabbit anti-Ago1 antibody (ab5070) [Abcam] and (B) the anti-Ago1 guinea pig antisera (BRD-GP-4). Blot was imaged with a ChemiDoc™ MP [BioRad]. (1) Eluate using immune sera from $w^{1118}$; P{w[+mC]=FLAG.HA.AGO2}2 flies. All other lanes without marker use lysate from $w^{1118}$ flies. (2) Eluate using preimmune antisera. (3) Marker. (4) Eluate using immune antisera. (5) Wash 5. (6) Wash 4. (7) Wash 3. (8) Wash 2. (9) Wash 1. (10) Supernatant from IP using immune antisera. (11) Input. (12) Marker.

**RI2 Fig. Full blot of Ago2 Immunoprecipitation used in Fig2C.** Immunoprecipitation (IP) using guinea pig antisera against Ago2 (BRD-GP-5) or preimmune antisera was conducted on lysate from whole male flies and probed using (A) a commercial rabbit anti-Ago2 antibody (ab5072) [Abcam] and (B) the anti-Ago2 guinea pig antisera (BRD-GP-6). Blot was imaged with a ChemiDoc™ MP [BioRad]. (1) Eluate using immune sera from $w^{1118}$; P{w[+mC]=FLAG.HA.AGO2}2 flies. All other lanes without marker use lysate from $w^{1118}$ flies. (2) Eluate using preimmune antisera. (3) Marker. (4) Eluate using immune antisera. (5) Wash 5. (6) Wash 4. (7) Wash 3. (8) Wash 2. (9) Wash 1. (10) Supernatant from IP using immune antisera. (11) Input. (12) Marker.

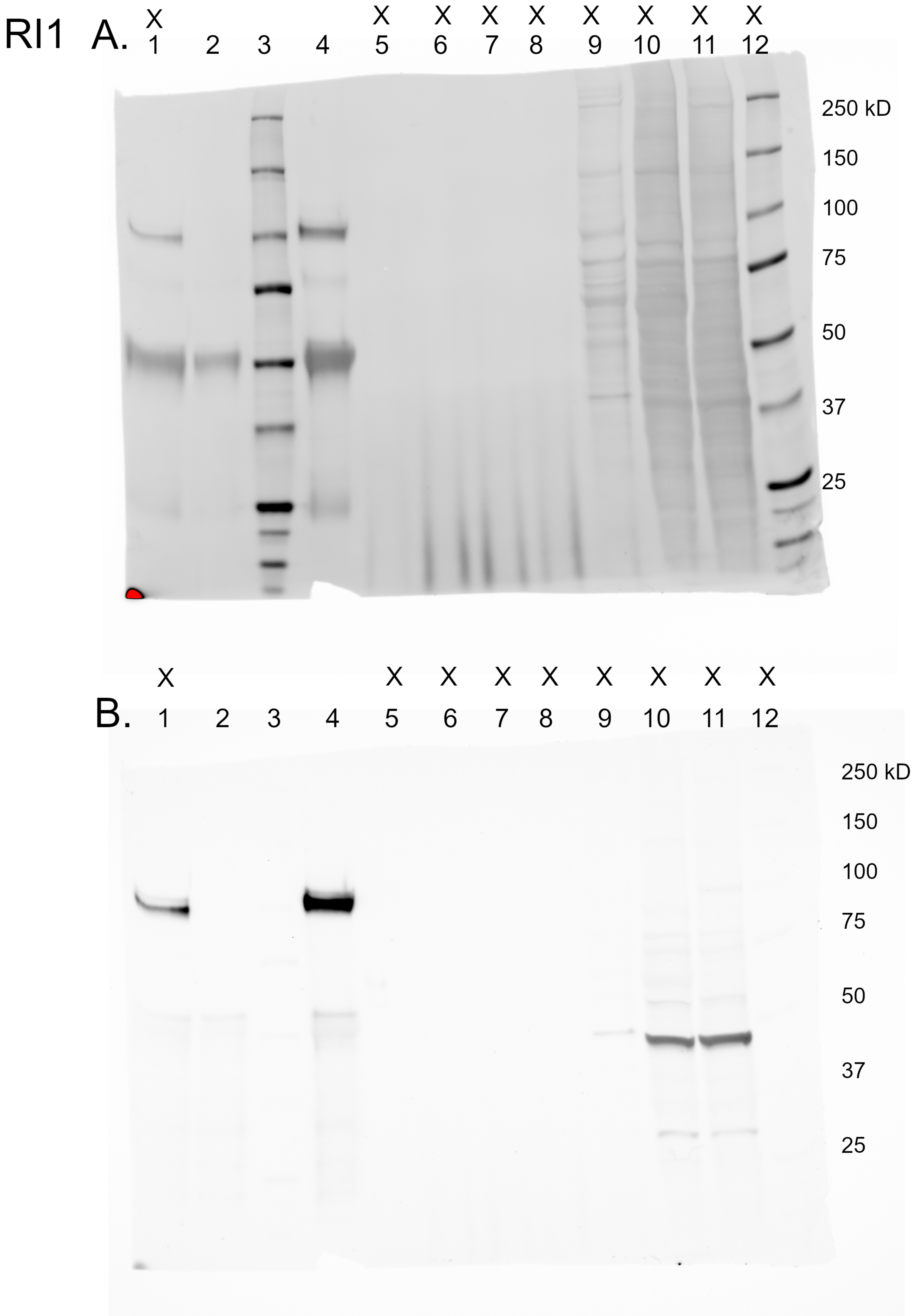
RI1 A.
X
1 2 3 4
X X X X X X X X
5 6 7 8 9 10 11 12
250 kD
150
100
75
50
37
25
B.
X
1 2 3 4
X X X X X X X X
5 6 7 8 9 10 11 12
250 kD
150
100
75
50
37
25

RI2

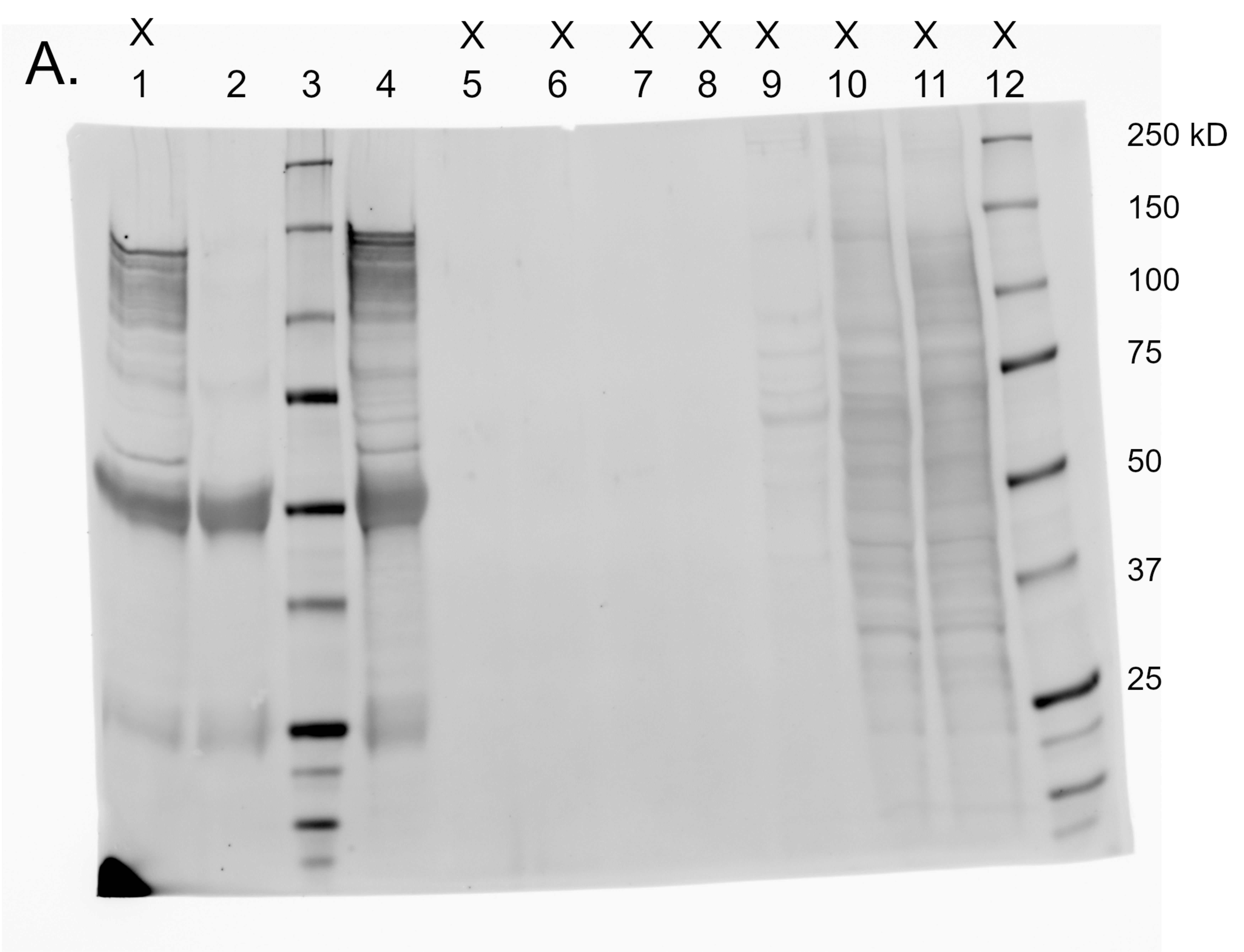
A.
X X X X X X X X X
1 2 3 4 5 6 7 8 9 10 11 12
250 kD
150
100
75
50
37
25

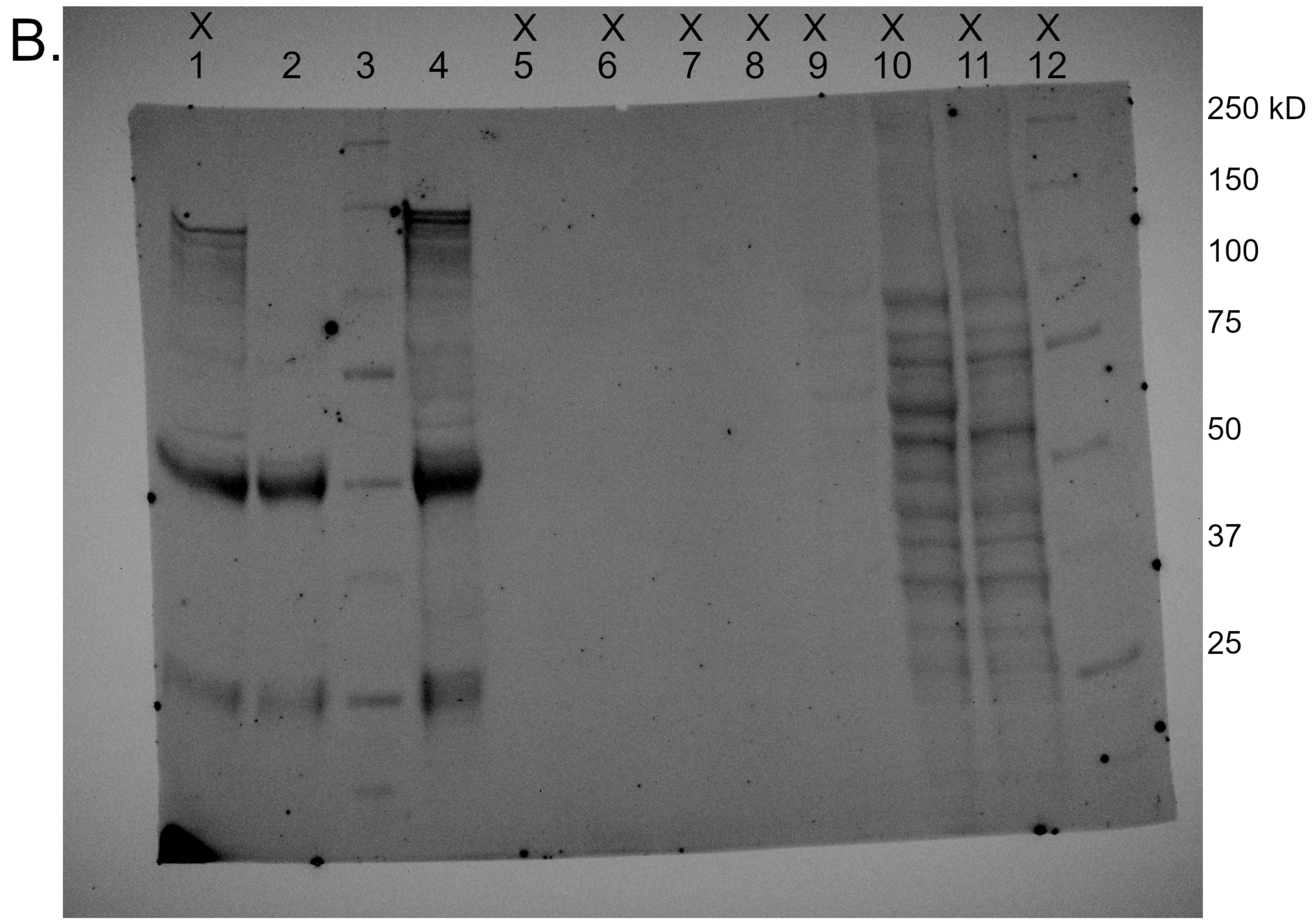
B.
X X X X X X X X X
1 2 3 4 5 6 7 8 9 10 11 12
250 kD
150
100
75
50
37
25

**RI3 Fig. Full blot of anti-Ago1 probe on Ago1 and Ago2 Immunoprecipitations used in S1A Fig.** Immunoprecipitations (IP) using guinea pig antisera against Ago1 (BRD-GP-3 and BRD-GP-4), guinea pig antisera against Ago2 (BRD-GP-5 and BRD-GP-6), or their matching preimmune sera were conducted on lysate from *Drosophila* S2 cells and probed using (A) a commercial rabbit anti-Ago1 antibody (ab5070) [Abcam] and (B) the anti-Ago1 guinea pig antisera (BRD-GP-3 and BRD-GP-4). Blot was imaged with a ChemiDoc™ MP [BioRad]. (1) Marker. (2) Input. (3) Supernatant from anti-Ago1 immune antisera IP. (4) Supernatant from anti-Ago1 preimmune sera IP. (5) Eluate from anti-Ago1 immune antisera IP. (6) Eluate from anti-Ago1 preimmune sera IP. (7) Marker. (8) Marker. (9) Supernatant from anti-Ago2 immune sera IP. (10) Supernatant from anti-Ago2 preimmune sera IP. (11) Eluate from anti-Ago2 immune antisera IP. (12) Eluate from anti-Ago2 preimmune sera IP.

**RI4 Fig. Full blot of anti-Ago2 probe on Ago1 and Ago2 Immunoprecipitations used in S1B Fig.** Immunoprecipitations (IP) using guinea pig antisera against Ago1 (BRD-GP-3 and BRD-GP-4), guinea pig antisera against Ago2 (BRD-GP-5 and BRD-GP-6), or their matching preimmune sera were conducted on lysate from *Drosophila* S2 cells and probed using (A) a commercial rabbit anti-Ago2 antibody (ab5072) [Abcam] and (B) the anti-Ago2 guinea pig antisera (BRD-GP-5 and BRD-GP-6). Blot was imaged with a ChemiDoc™ MP [BioRad]. (1) Marker. (2) Input. (3) Supernatant from anti-Ago1 immune antisera IP. (4) Supernatant from anti-Ago1 preimmune sera IP. (5) Eluate from anti-Ago1 immune antisera IP. (6) Eluate from anti-Ago1 preimmune sera IP. (7) Marker. (8) Marker. (9) Supernatant from anti-Ago2 immune sera IP. (10) Supernatant

from anti-Ago2 preimmune sera IP. (11) Eluate from anti-Ago2 immune antisera IP. (12) Eluate from anti-Ago2 preimmune sera IP.

RI3

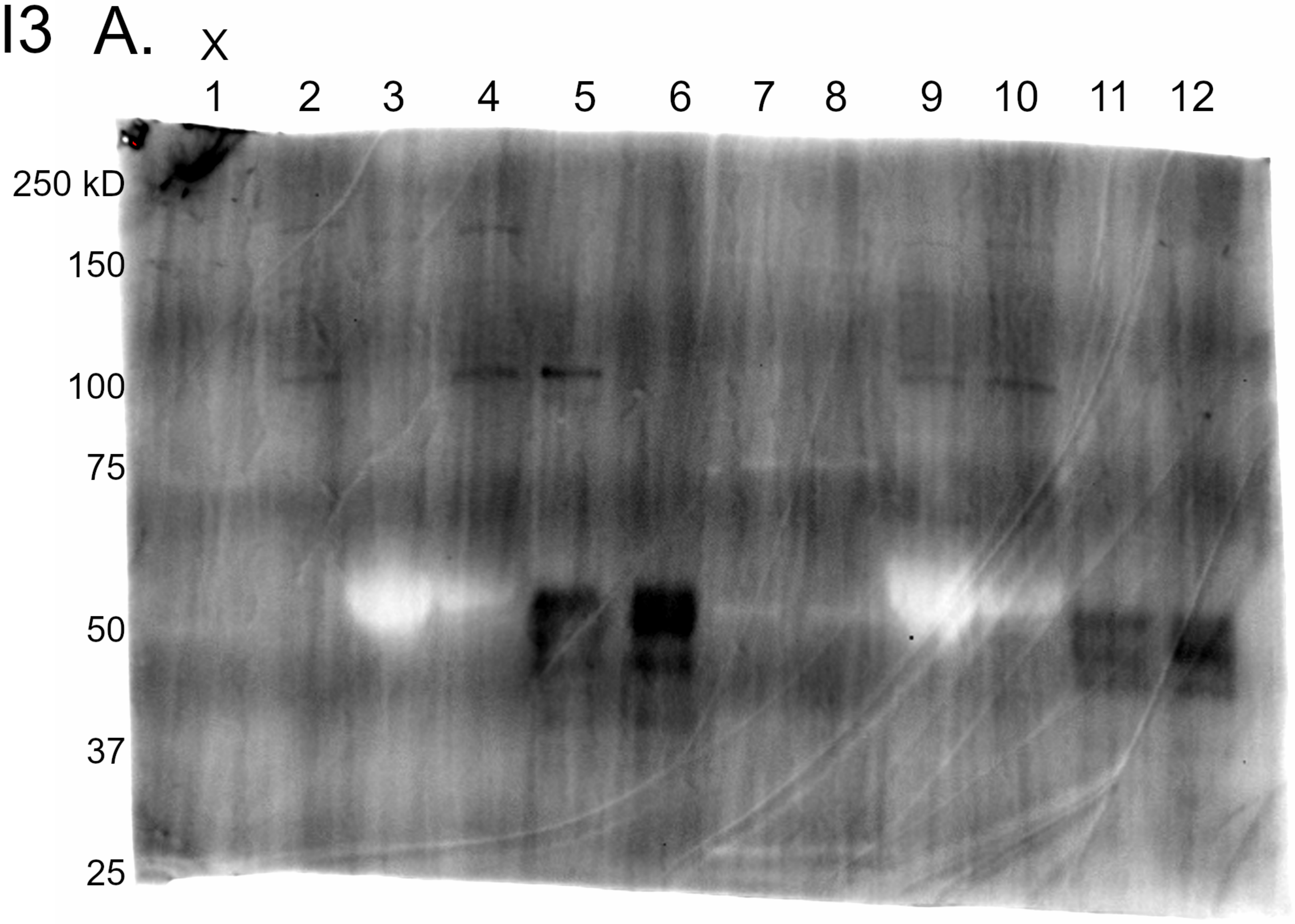
A.
X
1 2 3 4 5 6 7 8 9 10 11 12
250 kD
150
100
75
50
37
25

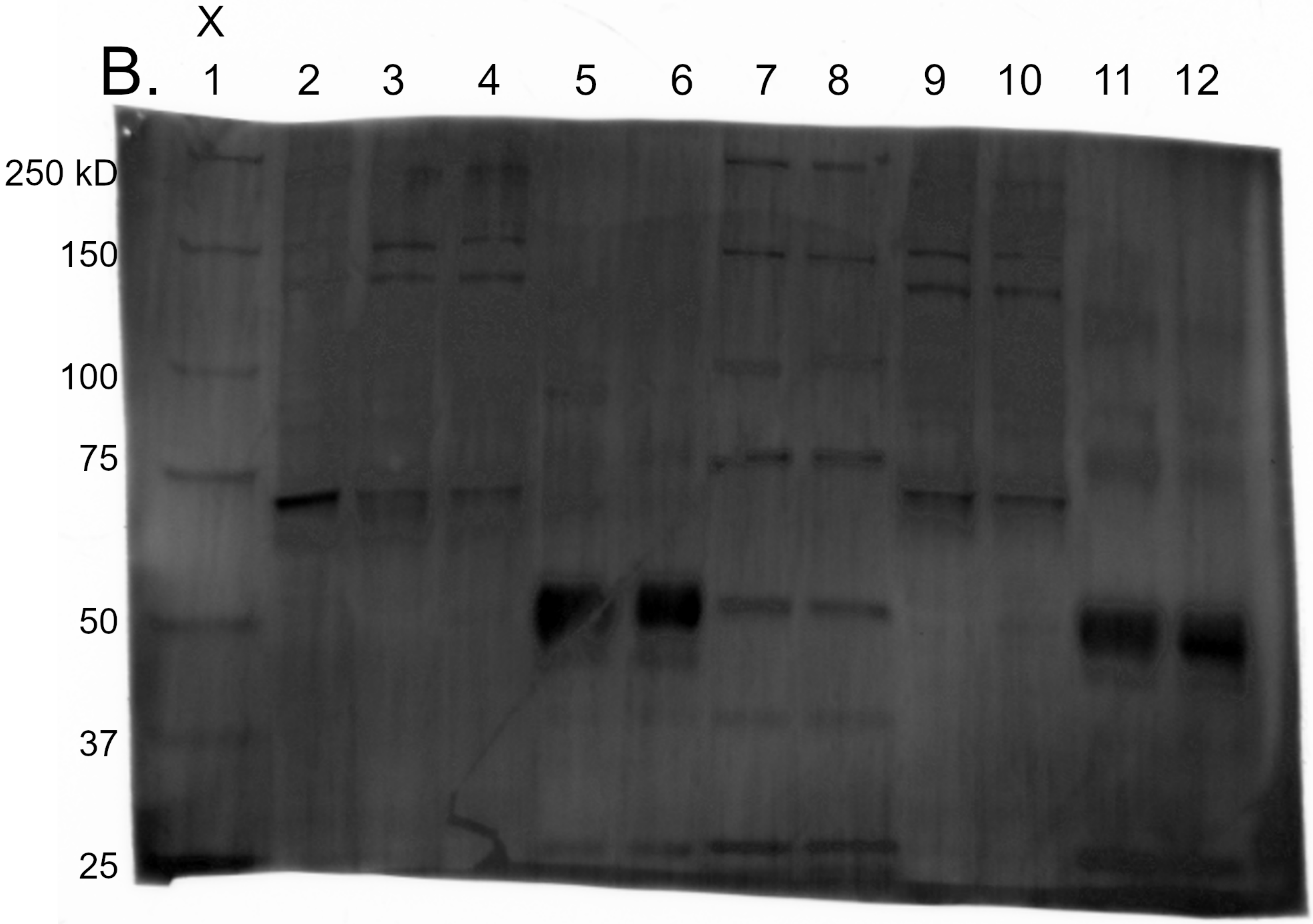
B.
X
1 2 3 4 5 6 7 8 9 10 11 12
250 kD
150
100
75
50
37
25

RI4 A.

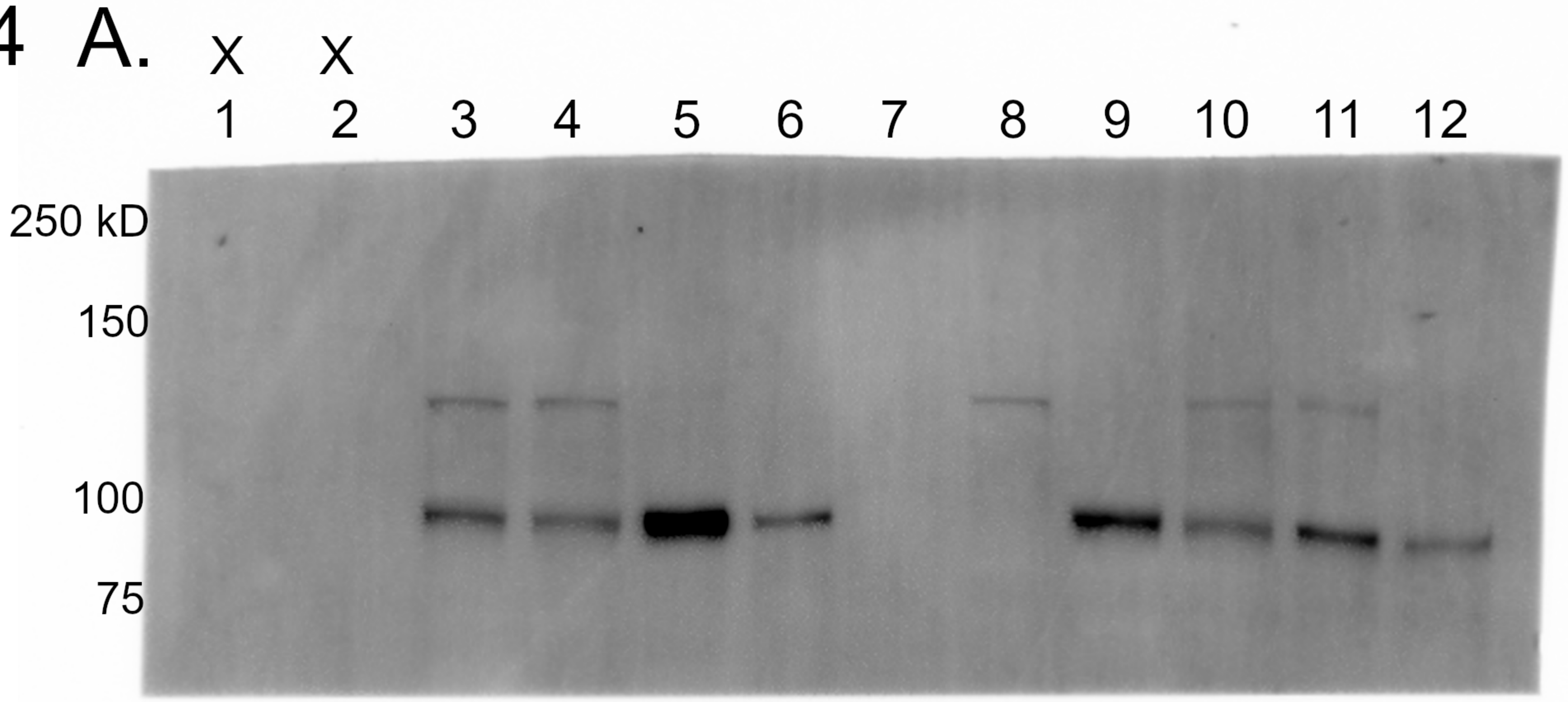
X
X
1
2
3
4
5
6
7
8
9
10
11
12
250 kD
150
100
75

B.

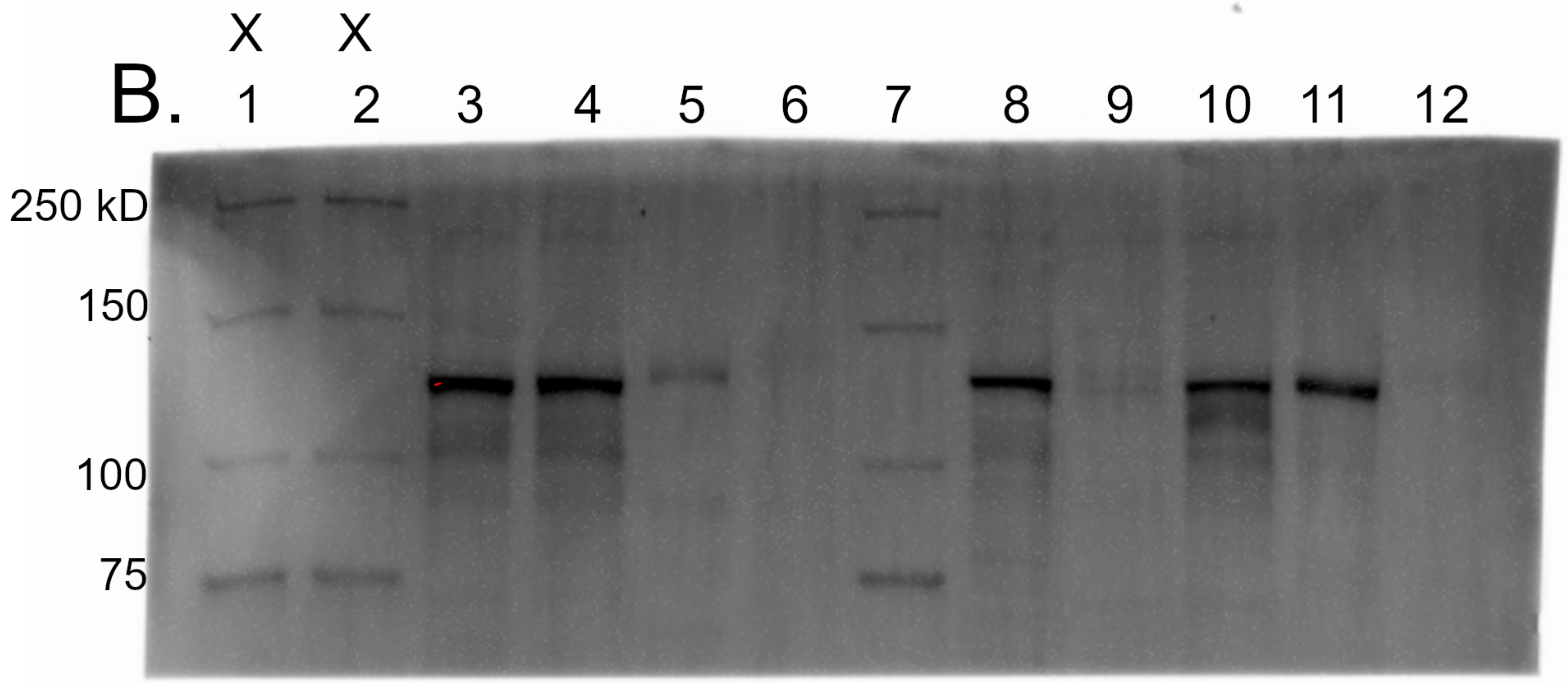
X
X
1
2
3
4
5
6
7
8
9
10
11
12
250 kD
150
100
75
